# Four-year real-world experience of secukinumab in a large Italian cohort of axial spondyloarthritis

**DOI:** 10.3389/fimmu.2024.1435599

**Published:** 2024-07-15

**Authors:** Roberta Ramonda, Mariagrazia Lorenzin, Maria Sole Chimenti, Salvatore D’Angelo, Antonio Marchesoni, Carlo Selmi, Ennio Lubrano, Leonardo Santo, Michele Maria Luchetti Gentiloni, Fabiola Atzeni, Alberto Cauli, Maria Manara, Maurizio Rossini, Roberta Foti, Giacomo Cozzi, Laura Scagnellato, Mario Ferraioli, Antonio Carriero, Nicoletta Luciano, Francesca Ruzzon, Mauro Fatica, Elena Fracassi, Andrea Doria, Rosario Foti, Antonio Carletto

**Affiliations:** ^1^ Rheumatology Unit, Department of Medicine DIMED, University of Padova, Padova, Italy; ^2^ Rheumatology, Allergology and Clinical Immunology, Department of Systems Medicine, University of Rome Tor Vergata, Rome, Italy; ^3^ Rheumatology Institute of Lucania (IReL), San Carlo Hospital of Potenza and Madonna delle Grazie Hospital of Matera, Potenza, Italy; ^4^ Rheumatology, Humanitas San Pio X, Milan, Italy; ^5^ Rheumatology and Clinical Immunology IRCCS Humanitas Research Hospital, Milan, Italy; ^6^ Department of Biomedical Sciences, Humanitas University, Milan, Italy; ^7^ Academic Rheumatology Unit, Dipartimento di Medicina e Scienze per la Salute “Vincenzo Tiberio”, University of Molise, Campobasso, Italy; ^8^ Rheumatology Unit, ASL BT Andria – DSS4, Barletta, Barletta-Andria-Trani, Italy; ^9^ Department of Clinical and Molecular Sciences, University Hospital of the Marche Region, Ancona, Italy; ^10^ Rheumatology Unit, University of Messina, Messina, Italy; ^11^ Rheumatology Unit, Department of Medical Sciences, AOU and University of Cagliari, Monserrato, Italy; ^12^ Department of Rheumatology, ASST Gaetano Pini-CTO, Milan, Italy; ^13^ Rheumatology Unit, Department of Medicine, AOUI University of Verona, Verona, Italy; ^14^ Rheumatology Unit, A.O.U. Policlinico S. Marco, Catania, Italy

**Keywords:** axial spondyloarthritis, r-axSpA/nr-axSpA biological therapy, secukinumab (IL17i), IL17i effectiveness, IL17i safety, IL17i drug retention rate

## Abstract

**Objectives:**

This study aims to evaluate in a real-life Italian multicenter cohort of axial spondyloarthritis (axSpA) (1) the 4-year effectiveness and safety of secukinumab, (2) the drug retention rate (DRR), and (3) the impact of the line of bDMARDs treatment, subtype of axSpA, and sex on achieving low disease activity (LDA) and very low disease activity (VLDA).

**Methods:**

Consecutive axSpA patients receiving secukinumab between 2016 and 2023 were prospectively evaluated. Data on disease characteristics, previous/ongoing treatments, comorbidities, and follow-up duration were collected. Treatment response was evaluated at 6 and 12 months after initiation and yearly up to 48 months (T48). DRR and effectiveness outcomes were evaluated according to bDMARDs treatment, axSpA subtype, and sex. Infections and adverse events (AEs) were recorded.

**Results:**

We enrolled 272 patients (48.2% male; median age, 51; 39.7% HLA-B27+; 40.4% nr-axSpA), of whom 30.9% were naïve to secukinumab. Overall, secukinumab yielded improvement in effectiveness outcomes; the naïve patients maintained lower disease activity vs. the non-naïve ones. At T48, the LDA and VLDA rates were higher in naïve patients and in male individuals. Treatment was discontinued in 104 patients due to primary/secondary loss of effectiveness and in 34 patients due to AEs. The DRR at T48 was 67.4% in the whole population, regardless of treatment line, axSpA subtype, and sex.

**Conclusions:**

Secukinumab was safe and effective in all axSpA patients irrespective of treatment line, disease subtype, and sex. The patients achieved sustained 4-year remission and DRR.

## Highlights:

What is already known about this subject?

• Secukinumab is a drug for axial spondyloarthritis (axSpA). Real-life long-term effectiveness and safety data is scarce, in particular with regard to the line of biological treatment (LoBT), axSpA subtype, and sex.

What does this study add?

• Our findings confirmed the safety and the remarkable effectiveness of secukinumab over a 48-month follow-up period.

• The drug retention rate (DRR) was considerably high at 48 months. The main clinical disease pattern (radiographic and non-radiographic involvement), sex, and LoBT did not influence the DRR of secukinumab over time.

• First-line bDMARDs and male sex appeared to correlate with higher low disease activity (LDA) and very low disease activity (VLDA) rates (measured via ASAS and BASDAI).

How might this impact on clinical practice?

• Our study supports the use of secukinumab as a treatment option even in patients who have undergone multi-drug failure. Furthermore, the safety of secukinumab allows its use in elderly patients or those with comorbidities, owing to the low prevalence of mild to moderate infections requiring drug discontinuation.

## 1 Introduction

Axial spondyloarthritis (axSpA) is a chronic inflammatory immune-mediated disease that belongs to the spectrum of spondyloarthritides (SpA). It predominantly affects the axial skeleton by causing inflammation and structural changes (1, 2) and can be classified into radiographic axSpA (r-axSpA) or non-radiographic axSpA (nr-axSpA), according to the presence of sacroiliitis on conventional radiographs in the former or the presence of active sacroiliitis on magnetic resonance imaging (MRI) in the absence of structural damage of sacroiliac joints (SIJ) detectable on X-rays in the latter (2). Patients suffer from pain, stiffness, restricted mobility, and functional deficits. The most typical symptom—inflammatory back pain (IBP)—is mainly caused by inflammation in the SIJ and/or the spine. Extraspinal manifestations (peripheral arthritis, enthesitis, and dactylitis) and extra-musculoskeletal manifestations such as anterior uveitis, psoriasis, and inflammatory bowel disease (IBD) may occur as well (1, 2). AxSpA is strongly associated with human leukocyte antigen (HLA)-B27 (3). The Assessment of SpondyloArthritis International Society (ASAS) classification criteria (4) are currently used for the classification of patients with axSpA. The *European Alliance of Associations for Rheumatology* (EULAR) and the *American College of Rheumatology* (ACR) guidelines recommend treating active axSpA patients with persistently high disease activity, despite undergoing non-steroidal anti-inflammatory drugs (NSAIDs), with biological disease-modifying anti-rheumatic drugs (bDMARDs) or targeted synthetic disease-modifying anti-rheumatic drugs (tsDMARDs) (5, 6). Tumor necrosis factor alpha inhibitors (TNFi) or IL-17 inhibitors (IL-17i) are now recommended as first-line bDMARD (7). Given that up to 50% of patients treated with TNFi do not achieve a clinically significant response, IL-17i have acquired a key role in axSpA treatment (8–11).

Secukinumab is the first-in-class human monoclonal IgG1κ antibody that directly inhibits IL-17A and is approved for the treatment of patients with axSpA (r-axSpA and nr-axSpA) (12). Secukinumab has demonstrated significant long-term efficacy and safety *versus* placebo across various randomized clinical trials (RCTs) in patients with axSpA who were either bDMARDs-naïve or had a history of treatment with TNFi (13–26).

Data from RCTs may not fully mimic secukinumab treatment in a real-world setting because clinical trials are highly regulated and do not represent everyday practice. Despite some limitations associated with real-world evidence (RWE) studies, such as analysis design and incomplete or missing data, these observational studies (prospective or retrospective) complement the evidence generated by RCTs and depend on everyday therapeutic use in the real-world setting.

In the context of axSpA, other RWE studies have investigated the effectiveness and safety of secukinumab in a real-life setting both in Italian (27–30) and international cohorts (31–36), even in comparison with TNFi (9, 10, 31, 32), but only for a limited observational period. In addition, the impact of different lines of bDMARDs treatment (LoBT), axSpA subtype, and sex on the achievement of clinical remission and on secukinumab drug survival has not yet been fully investigated (28, 29). Establishing secukinumab’s effectiveness, safety, and drug retention rate is key to improve treatment decision-making.

Our prospective observational study aimed to evaluate, in an Italian multicenter, real-life cohort of axSpA patients on secukinumab followed for 48 months, (1) the long-term effectiveness and safety, (2) the drug retention rate (DRR) and the reasons for discontinuation, and (3) the impact of LoBT (naïve/non-naïve), axSpA subtype [radiographic-axSpA (r-axSpA)/non-radiographic-axSpA (nr-axSpA)], and sex (male/female) on achieving low disease activity (LDA), measured as Bath Ankylosing Spondylitis Disease Activity Index (BASDAI) <4/Ankylosing Spondylitis Disease Activity Score (ASDAS) <2.1, and very low disease activity (VLDA), measured as BASDAI <2/ASDAS <1.3.

## 2 Materials and methods

### 2.1 Study design, patients, and data collection

A cohort of patients with axSpA treated with secukinumab were studied in an observational prospective study from September 2016 to May 2023 in 11 Italian rheumatology centers. The study was sustained by the Italian Society of Rheumatology (SIR) “Spondyloarthritis and Psoriatic Arthritis study group—A. Spadaro.”

Patients’ written consents were acquired according to the Declaration of Helsinki, when their data were recorded in the database for treatment. The Ethics Committee’s approval was obtained from all participating centers (approval no. 23943), as well as the written informed consent for the anonymous use of personal data from every patient, in compliance with Italian Legislative Decree 196/2003.

All included patients had undergone structured medical and physical examinations by rheumatologists. Demographic data were collected, including age, sex, body mass index (BMI), disease characteristics, duration and onset of back pain, whether IBP was present, age at diagnosis, history and/or presence of arthritis, enthesitis, dactylitis, and extra-articular SpA manifestations (anterior uveitis, psoriasis, and IBD), response to NSAIDs, and family history of SpA. In addition, C-reactive protein (CRP), erythrocyte sedimentation rate (ESR) level, and HLA-B27 were determined, and X-rays and magnetic resonance imaging (MRI) of the SIJ was performed if clinically justified. Treatment data were collected about previous/ongoing treatments, concomitant medications, including conventional synthetic (cs) disease-modifying antirheumatic drugs (DMARDs), NSAIDs, and glucocorticosteroids (GCs), or previous biologics at the time of the first administration of secukinumab. The presence of comorbidities and concomitant therapies was also investigated (yes/no) and registered. Baseline data were retrieved by reviewing the clinical charts, face-to-face interview, and patients’ extensive medical record.

### 2.2 Patients’ enrollment and follow-up

Axial SpA was diagnosed using ASAS criteria (4), and patients who initiated secukinumab treatment for moderate or severe disease according to EULAR and ACR guidelines were considered (5, 6), and those who continued taking secukinumab for more than three months were included. All patients were screened before enrollment and starting treatment as suggested by the European guidelines (5, 6). In Europe, the recommended starting dose in r-axSpA and nr-axSpA is 150 mg once weekly for the first five doses and then 150 mg per month thereafter, in accordance with the manufacturer’s instructions (22). In subjects affected by active and severe psoriasis—according to the treating physician—a dosage up to 300 mg per administration could be employed. Follow-up started at the start date of secukinumab administration and ended at the stop date for the treatment, death, or the end of the study (May 31, 2023), whichever occurred first. Finally, the duration of secukinumab therapy (expressed in months), previous LoBT, reasons for discontinuation (e.g., ineffectiveness, side effects, adverse events—AEs), infections, concomitant GCs, csDMARDs, and NSAIDs were also registered in our cohort of patients.

### 2.3 Treatment response

Initially, the treatment response was assessed at 6 and 12 months, followed by annual assessment until 48 months.

Effectiveness outcomes: Relevant Patient Reported Outcomes (PROs) (37), Visual Analogue Scale of pain (VAS-pain) and global health (VAS-GH) and physician’s assessment (VAS-Ph), Health Assessment Questionnaires modified for spondyloarthritis (HAQ-S), Bath Ankylosing Spondylitis Functional Index (BASFI), and Bath Ankylosing Spondylitis Disease Activity Index (BASDAI) were collected in the whole cohort. Bath Ankylosing Spondylitis Metrology Index (BASMI), Leeds Enthesitis Index (LEI), and Ankylosing Spondylitis Disease Activity Score (ASDAS) were recorded by an experienced rheumatologist at each assessment (37). Erythrocyte sedimentation rate (ESR) and the C-reactive protein (CRP) value were considered and evaluated (ESR 0–25 mm/h; CRP 0–6 mg/L normal range).

Disease activity composite measures: LDA and VLDA at T6, T12, T24, T36, and T48 according to BASDAI and ASDAS (BASDAI-LDA defined as <4, BASDAI-VLDA defined as <2, ASDAS-LDA defined as <2.1, ASDAS-VLDA defined as <1.3) were assessed (37). The AxSpA patients were divided in two subgroups (r-axSpA/nr-axSpA) according to the LoBT (naïve vs. non-naïve patients) and in different sex (male/female) to calculate LDA and VLDA.

### 2.4 Treatment retention

Drug survival of up to 4 years of secukinumab treatment was defined as the probability of long-term drug retention rate (DRR), as shown by Kaplan–Meier curves. It was calculated as the number of days of treatment (start and stop date from the first and last dose of treatment).

### 2.5 Statistical analysis

Statistics firstly provided a descriptive analysis of the collected data. Data were expressed as frequencies and percentages for categorical variables and as median and interquartile range (IQR) for continuous variables. Naïve and non-naïve patients’ characteristics were compared using the chi-square test or the Fisher exact test for categorical variables and the *t*-test or the Wilcoxon rank test for continuous variables, based on data distribution. Effectiveness measures and outcomes data were compared from the beginning and after 48 months with the chi-square test or the Wilcoxon rank test as appropriate. The Kaplan–Meier curve was exploited to measure the cumulative DRR of secukinumab considering discontinuation due to ineffectiveness or AEs. Furthermore, Kaplan–Meier curves were also retained to assess the impact of previous LoBT, r-axSpA/nr-axSpA, sex, and patients’ clinical characteristics on the DRR of secukinumab. Survival curves were completed by using the log rank test. All statistical analyses were carried out with the SPSS 13.0 software (SSPS Inc., IL, USA). Two-tailed *p*-values lower than 0.05 were considered statistically significant.

## 3 Results

### 3.1 Patients’ characteristics

A total of 272 axSpA patients were evaluated [48.2% male; median age, 51 (41–59); 39.7% HLA-B27+; 40.4% nr-axSpA] with median disease duration of 9 years and median treatment duration of 40 (14–57) months. In 84 patients (30.9%), secukinumab was the first-line biological treatment (naïve) and in 188 patients (69.1%) the second-line (or more) biological treatment (non-naïve). The following extra-articular manifestations were recorded: psoriasis (38.9%, *n* = 106), onychopathy (21%, *n* = 57), IBD (5.9%, *n* = 16) in remission, and uveitis (9.2%, *n* = 25) in remission. The baseline (T0) characteristics as per clinical and laboratory investigations, such as simultaneous treatments, are summarized in **Table 1**. At T0, non-naïve (*versus* naïve) patients were older and more frequently HLA-B27-positive, had a longer duration of axial symptoms, higher signs of active or radiographic sacroiliitis, had a greater prevalence of peripheral arthritis, enthesitis, dactylitis, and psoriasis, and worse functional status (HAQ-S and BASFI) and disease activity indices (higher CRP and BASDAI values) (**Table 1**). The extra-articular features (IBD and uveitis) and the other clinical and functional parameters did not show significant differences (**Table 1**).

**Table 1:** Baseline characteristics of 272 axSpA patients treated with secukinumab during 48 months of follow-up.

	Total, patients	Naïve vs.	Non-naïve	p^§^
AxSpA features				
Male sex (*N*, %)	131 (48.2)	41 (48.8)	90 (47.9)	ns
Age (years), median (IQR)	51 (41–59)	46 (36–58)	53 (43–60)	ns
Age at diagnosis (years), median (IQR)	42 (31–51)	39.5 (29–50)	42 (34–51)	0.04
Age at disease onset (years), median (IQR)	39 (29–50)	35.5 (26–49)	40 (31–50)	ns
Disease duration (years), median (IQR)	9 (4–14)	7 (3–14)	9 (5–14)	0.04
axSpA	272	84	188	N/A
r-axSpA, *N (%)*	162 (59.6)	43 (51.2)	119 (63.3)	0.03
nr-axSpA, *N* (%)	110 (40.4)	41 (48.8)	69 (36.7)	0.03
HLA-B27-positive, *N* (%)	108 (39.7)	40 (47.6)	68 (36.2)	0.04
SIJ-MRI-positive, *N* (%)	210 (77.2)	61 (72.6)	149 (79.3)	0.04
SIJ-X-rays-positive, *N* (%)	130 (47.8)	39 (46.4)	91 (48.4)	0.04
Peripheral arthritis, *N* (%)	150 (55.1)	35 (41.7)	115 (61.2)	0.02
Enthesitis, *N* (%)	107 (39.3)	29 (34.5)	78 (41.4)	0.03
Dactylitis, *N* (%)	30 (11.0)	8 (9.5)	22 (11.7)	0.04
Psoriasis, *N* (%)	106 (38.9)	32 (38.1)	74 (39.4)	0.049
Onychopathy, *N* (%)	57 (21.0)	16 (19.1)	41 (21.8)	0.04
IBD in remission, *N* (%)	16 (5.9)	5 (5.9)	11 (5.9)	ns
Uveitis in remission, *N* (%)	25 (9.2)	8 (9.5)	17 (9.1)	ns
Familiarity with psoriasis or SpA, *N* (%)	83 (30.5)	25 (29.8)	58 (30.9)	ns
Smoking, *N* (%)	87 (31.9)	26 (31.0)	61 (32.4)	ns
Weight (kg), median (IQR)	72.5 (63–82)	74.5 (66.5–80.3)	72 (62–82)	ns
Height (cm), median (IQR)	170 (161.5–176.5)	173.5 (163.5–176.3)	168 (161–176.5)	ns
BMI, median (IQR)	25.1 (22.5–28.1)	24.6 (23.1–27.9)	25.4 (22.2–28.1)	ns
Clinical, serological, and functional indices				
BASMI [0–10], median (IQR)	3 (1–6)	2 (1–5)	2.5 (1–6)	ns
LEI [0–6], median (IQR)	1 (0–2)	1 (0–1.5)	1 (0–2)	ns
ESR [0–25] (mm/h), median (IQR)	15 (10–27)	15 (7.5–21.8)	16 (10–29)	ns
CRP [0–6] (mg/L), median (IQR)	3.4 (2.9–8.1)	3.2 (2.0–8.0)	4 (2.1–9.7)	0.04
VAS-pain [0–10], median (IQR)	7 (5–8)	6 (5–8)	7 (5.5–8)	ns
VAS-GH [0–10], median (IQR)	5.5 (4–7)	5 (4–7)	6 (4–7)	ns
VAS-Ph [0–10], median (IQR)	6 (5–7.8)	6 (5–7)	6.7 (5–8)	ns
HAQ-S [0–8], median (IQR)	1 (0.5–1.5)	0.6 (0.3–1.1)	1.1 (0.5–1.5)	0.048
BASFI [0–10], median (IQR)	6 (4.5–7.0)	5.5 (4.0–6.5)	6.2 (5.1–7.4)	0.04
BASDAI [0–10], median (IQR)	6.4 (4.8–7.5)	5.7 (4.5–6.8)	6.5 (5.1–7.8)	0.04
ASDAS [0–6], median (IQR)	3.3 (2.6–3.5)	3.0 (2.8–3.4)	3.2 (2.6–3.6)	ns
Treatment				
Treatment duration (months), median (IQR)	40 (14–57)	42 (16–55.5)	43 (17–56.5)	ns
Dosage 300 mg/injection, *N* (%)	68 (25.0)	9 (10.7)	59 (31.4)	0.04
Dosage 150 mg/injection, *N* (%)	204 (75.0)	75 (89.3)	129 (68.6)	0.04
1st line, *N* (%)	84 (30.9)	84 (100)	0 (0)	N/A
Failure biological drugs, *N* (%)	188 (69.1)	0 (0)	188 (100)	N/A
2nd line, *N* (%)	84 (28.3)	0 (0)	84 (28.3)	N/A
3rd line, *N* (%)	57 (20.9)	0 (0)	57 (20.9)	N/A
4th line, *N* (%)	33 (12.1)	0 (0)	33 (12.1)	N/A
>5th line, *N* (%)	14 (5.1)	0 (0)	14 (5.1)	N/A
Concomitant NSAIDs, *N* (%)	160 (58.8)	46 (54.5)	114 (60.6)	ns
Concomitant glucocorticoids, *N* (%)	36 (13.2)	10 (11.9)	26 (13.8)	ns
Concomitant csDMARDs, *N* (%)	45 (16.5)	11 (13.1)	34 (18.1)	ns

Data are expressed as median (interquartile range = IQR) or number (percentage = %) unless otherwise specified; the range of possible values is indicated in round brackets. p^§^, chi-square test or Fisher’s exact test for categorical variables and t-test or Wilcoxon rank test for continuous variables at T0: p <0.05.

naïve = naïve to anti-TNF alpha inhibitors; non-naïve = TNF alpha failure inhibitors; SpA, spondyloarthritis; axSpA, axial spondyloarthritis; r-axSpA, radiographic axial spondyloarthritis; nr-axSpA, non-radiographic axial spondyloarthritis; IBD, inflammatory bowel disease; HLA-B27, human leukocyte antigen (HLA) B27; SIJ, sacroiliac joint; MRI, magnetic resonance imaging; X-rays; conventional radiography; Kg, kilogram; cm, centimeter; BMI, body mass index; BASMI, Bath Ankylosing Spondylitis Metrology Index; LEI, Leeds Enthesitis Index; ESR, erythrocyte sedimentation rate; CRP, C-reactive protein; VAS, Visual Analogue Scale; GH, global health; Ph, physician’s assessment; BASDAI, Bath Ankylosing Spondylitis Disease Activity Index; BASFI, Bath Ankylosing Spondylitis Functional Index; ASDAS, Ankylosing Spondylitis Disease Activity Score; HAQ-S, Health Assessment Questionnaire modified for spondyloarthritis; NSAIDs, non-steroidal inflammatory drugs; csDMARDs, conventional synthetic disease-modifying antirheumatic drugs; ns, not statistically significant; N/A, not applicable.

### 3.2 Therapy effectiveness

There was a total of 272 axSpA patients, of which 256 (94.1%) were assessed at T6, 235 (86.4%) at T12, 191 (70.2%) at T24, 154 (56.6%) at T36, and 90 (33.1%) at T48.

A significant decrease in VAS pain (*p* = 0.01), VAS-GH (*p* = 0.03), VAS-Ph (*p* = 0.02), BASMI (*p* = 0.03), LEI (*p* = 0.04), HAQ-S (*p* = 0.04), BASFI (*p* = 0.03), ESR (*p* = 0.04), and CRP (*p* = 0.04) (**Table 2**) was observed in all patients. The ASDAS [T0 = 3.1 (2.7–3.5) vs. T48 = 1.2 (0.9–2.1); *p* = 0.02)] and BASDAI [T0 = 6.4 (4.8–7.5) vs. T48 = 2.0 (1.1–3.4); *p* = 0.03)] scores were significantly improved. At T48, the naïve patients showed better physical function and lower inflammatory activity vs. the non-naïve patients [ASDAS naïve vs. non-naïve = 1.1 (0.9–1.7) vs. 1.4 (1.1–1.9) (*p* = 0.02); BASDAI naïve vs. non-naïve = 1.6 (0.6–2.2) vs. 2.0 (1.0–3.5) (*p* = 0.03)] (**Table 2**).

**Table 2:** Clinical, functional, disease activity, and serological parameters of all (*n* = 272), naïve (*n* = 84), and non-naïve (*n* = 188) axSpA patients during the 48-month follow-up.

	T0	T6	T12	T24	T36	T48	*P* ^§^
BASMI [0–10], median (IQR)	3.0 (1.0–6.0)	3.0 (1.0–5.0)	2.0 (0.0–4.0)	1.5 (0.0–3.0)	1.0 (0.0–2.0)	1.0 (0.0–2.0)	0.03
Naïve	2.0 (1.0–5.0)	2.0 (1.0–3.3)	1.0 (0.0–2.2)	1.0 (0.0–2.0)	1.0 (0.0–1.0)	1.0 (0.0–1.0)	
Non-naïve	2.5 (1.0–6.0)	3.0 (1.0–6.0)	2.0 (1.0–4.0)	1.0 (0.0–3.0)	1.0 (0.0–2.0)	1.0 (0.0–1.8)	
p*	ns	0.05	0.05	ns	ns	ns	
LEI [0–6], median (IQR)	1.0 (0.0–2.0)	1.0 (0.0–1.0)	0.0 (0.0–0.0)	0.0 (0.0–0.0)	0.0 (0.0–0.0)	0.0 (0.0–0.0)	0.04
Naïve	1.0 (0.0–1.5)	0.1 (0.0–1.0)	0.0 (0.0–0.0)	0.0 (0.0–0.0)	0.0 (0.0–0.0)	0.0 (0.0–0.0)	
Non-naïve	1.0 (0.0–2.0)	1.0 (0.0–2.0)	0.0 (0.0–0.0)	0.0 (0.0–0.0)	0.0 (0.0–0.0)	0.0 (0.0–0.0)	
p*	ns	ns	ns	ns	ns	ns	
VAS pain [0–10], median (IQR)	7.0 (5.0–8.0)	5.0 (2.0–4.0)	3.0 (2.0–5.0)	3.0 (1.0–4.0)	2.0 (1.0–4.0)	2.0 (1.0–3.0)	0.01
Naïve	6.0 (5.0–8.0)	4.0 (2.0–5.0)	2.2 (2.0–4.7)	2.0 (1.0–3.0)	1.0 (1.0–3.0)	1.0 (0.0–3.0)	
Non-naïve	7.0 (5.5–8.0)	5.0 (2.0–6.0)	3.0 (2.0–5.0)	3.0 (1.0–5.0)	2.0 (1.0–4.0)	2.0 (1.0–3.0)	
p*	ns	ns	ns	ns	ns	ns	
VAS-GH [0–10], median (IQR)	5.5 (4.0–7.0)	5.0 (3.0–6.0)	3.5 (2.0–5.4)	3.0 (1.5–5.0)	2.0 (1.0–5.0)	2.0 (1.0–4.0)	0.03
Naïve	5.0 (4.0–7.0)	4.0 (2.0–6.0)	3.0 (2.0–5.5)	2.0 (1.0–4.0)	2.0 (1.0–4.0)	1.0 (0.0–3.0)	
Non-naïve	6.0 (4.0–7.0)	5.5 (3.0–6.0)	4.0 (3.0–6.0)	3.0 (2.0–5.0)	2.0 (1.0–5.0)	2.0 (1.0–4.0)	
p*	ns	0.05	ns	ns	ns	0.05	
VAS-Ph [0–10], median (IQR)	6.0 (5.0–7.8)	5.0 (4.0–6.0)	3.2 (2.0–4.0)	3.0 (2.0–4.0)	2.0 (1.0–3.0)	2.0 (1.0–3.0)	0.02
Naïve	6.0 (5.0–7.0)	4.0 (2.0–5.0)	3.0 (1.0–3.0)	2.0 (0.0–3.0)	1.0 (0.0–3.0)	1.0 (0.0–3.0)	
Non-naïve	6.7 (5.0–8.0)	5.0 (3.0–6.0)	3.0 (2.0–4.0)	2.0 (1.0–4.0)	2.0 (1.0–4.0)	2.0 (1.0–3.0)	
p*	ns	ns	ns	ns	ns	ns	
HAQ-S [0–8], median (IQR)	1.0 (0.5–1.5)	0.8 (0.3–1.2)	0.5 (0.2–1.1)	0.4 (0.1–1.1)	0.4 (0.1–1.0)	0.3 (0.1–1.0)	0.04
Naïve	0.6 (0.3–1.1)	0.5 (0.2–1.0)	0.3 (0.0–1.1)	0.3 (0.0–0.5)	0.2 (0.0–0.5)	0.1 (0.0–0.5)	
Non-naïve	1.1 (0.5–1.5)	0.8 (0.5–1.3)	0.8 (0.3–1.2)	0.5 (0.1–1.1)	0.6 (0.1–1.0)	0.5 (0.1–1.0)	
p*	0.05	ns	ns	ns	ns	ns	
BASFI [0–10], median (IQR)	6.0 (4.5–7.0)	4.4 (2.4–5.4)	3.4 (2.0–5.0)	2.1 (1.9–4.6)	2.0 (1.5–4.1)	2.0 (1.2–4.0)	0.03
Naïve	5.5 (4.0–6.5)	3.8 (1.9–5.3)	3.0 (1.5–4.0)	2.0 (1.4–3.5)	2.0 (1.0–3.0)	2.0 (1.0–4.0)	
Non-naïve	6.2 (5.1–7.4)	4.3 (2.7–5.3)	3.0 (2.0–5.0)	3.0 (2.0–5.0)	3.0 (1.0–5.0)	2.8 (1.9–4.5)	
p*	0.04	0.04	0.04	0.04	0.05	0.05	
BASDAI [0–10], median (IQR)	6.4 (4.8–7.5)	4.2 (2.7–6.1)	3.4 (2.1–5.0)	2.5 (1.8–4.5)	2.2 (1.6–3.8)	1.8 (1.2–3.3)	0.03
Naïve	5.7 (4.5–6.8)	3.9 (2.0–5.0)	2.8 (1.5–4.5)	2.0 (0.8–3.2)	1.4 (0.6–2.8)	1.6 (0.6–2.2)	
Non-naïve	6.5 (5.1–7.8)	4.5 (2.6–6.2)	3.2 (2.2–5.3)	3.0 (2.0–4.9)	3.0 (1.2–4.5)	2.0 (1.0–3.5)	
p*	0.04	0.04	0.04	0.04	0.03	0.04	
ASDAS [0–6], median (IQR)	3.3 (2.6–3.5)	2.8 (2.1–3.5)	2.2 (1.6–3.2)	2.0 (1.3–2.9)	1.8 (1.1–2.3)	1.2 (0.9–1.9)	0.02
Naïve	3.0 (2.8–3.4)	2.3 (1.4–3.2)	2.1 (1.4–3.0)	1.5 (1.0–2.6)	1.2 (0.9–1.9)	1.1 (0.9–1.7)	
Non-naïve	3.3 (2.6–3.6)	2.8 (1.9–3.6)	2.4 (1.7–3.2)	2.2 (1.5–3.0)	1.9 (1.2–2.6)	1.4 (1.1–1.9)	
p*	ns	0.05	0.05	0.05	0.05	ns	
ESR [0–25] (mm/h), median (IQR)	15.0 (10.0–27.0)	12.0 (6.0–20.5)	10.0 (5.0–18.0)	9.0 (4.0–16.5)	8.0 (4.0–15.5)	7.0 (4.0–13.0)	0.04
Naïve	15.0 (7.5–21.8)	10.0 (4.0–20.0)	7.0 (3.0–12.0)	7.0 (3.0–10.5)	6.0 (4.0–15.0)	8.0 (4.0–12.5)	
Non-naïve	16.0 (10.0–29.0)	13.0 (7.0–20.0)	12.0 (6.0–21.0)	11.0 (5.0–19)	9.0 (4.2–16.0)	9.0 (4.0–16.0)	
p*	ns	0.05	0.05	0.04	0.04	0.05	
CRP [0–6] (mg/L), median (IQR)	3.4 (2.9–8.1)	2.9 (1.5–5.2)	2.6 (1.0–4.9)	2.0 (1.0–3.8)	2.0 (1.0–4.0)	2.0 (1.0–3.0)	0.04
Naïve	3.2 (2.0–8.0)	2.4 (1.0–4.0)	2.2 (1.0–3.5)	2.0 (1.0–3.0)	2.0 (1.0–3.0)	2.0 (1.0–3.0)	
Non-naïve	4.0 (2.1–9.7)	2.9 (1.4–5.5)	2.9 (1.2–5.0)	2.4 (1.0–4.8)	2.5 (1.0–4.0)	2.0 (1.0–3.0)	
p*	0.04	0.05	0.04	ns	ns	ns	

Data are expressed as median (interquartile range = IQR). p < 0.05. Values were computed by means of Wilcoxon’s test (for continuous data). P^§^ < 0.05, T48 vs. T0. p* < 0.05, naïve vs. non-naïve.

BASMI, Bath Ankylosing Spondylitis Metrology Index; LEI, Leeds Enthesitis Index; VAS pain, Visual Analogue Scale pain; VAS GH, Visual Analogue Scale global health; VAS Ph, Visual Analogue Scale physician’s assessment; HAQ, Health Assessment Questionnaire modified for spondyloarthritis; BASFI, Bath Ankylosing Spondylitis Functional Index; BASDAI: Bath Ankylosing Spondylitis Disease Activity Index; ASDAS, Ankylosing Spondylitis Disease Activity Score; ESR, erythrocyte sedimentation rate; CRP, C-reactive protein.

**Figure 1:**
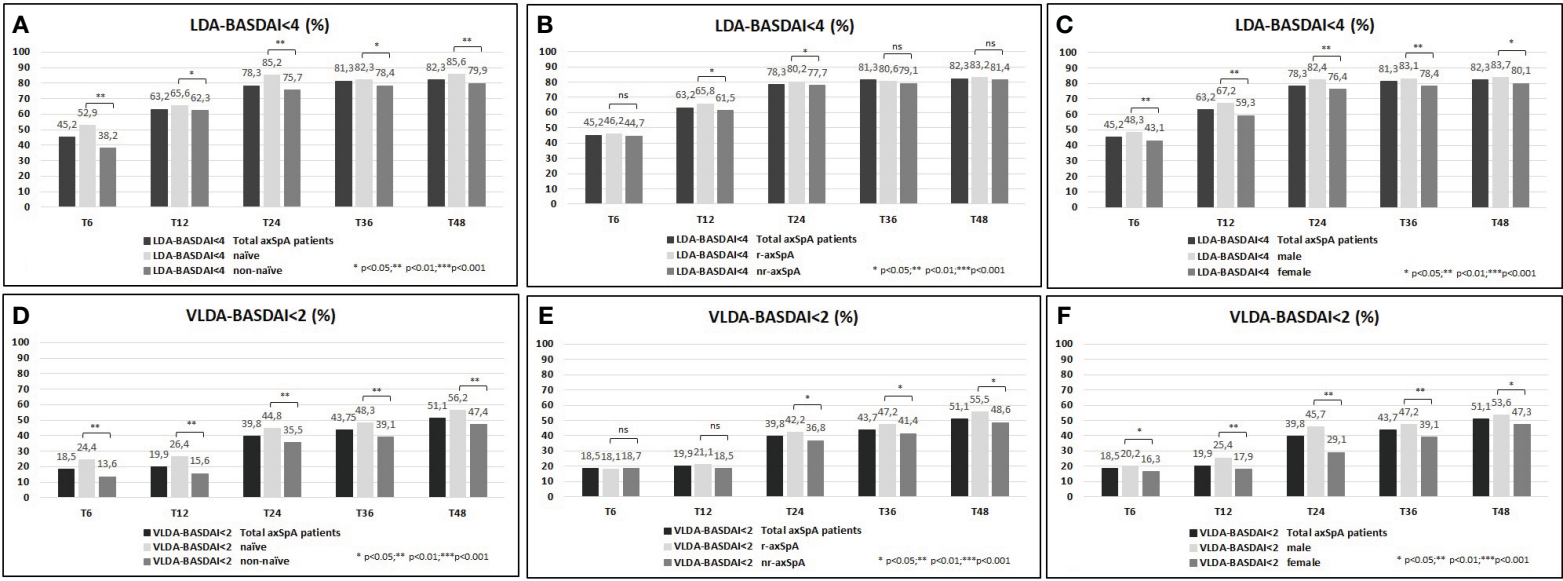
**(A–F)** BASDAI <4 (percentage, %) of the overall study population and after their subdivision in two groups according to lines of biological treatment (LoBT) (naïve/non-naïve) **(A)**, axSpA subtype (r-axSpA/nr-axSpA) **(B)**, and sex (male/female) **(C)**. BASDAI <2 (percentage, %) of the overall study population and after their subdivision in two groups according to lines of biological treatment (LoBT) (naïve/non-naïve) **(D)**, axSpA subtype (r-axSpA/nr-axSpA) **(E)**, and sex (male/female) **(F)**.

**Figure 2:**
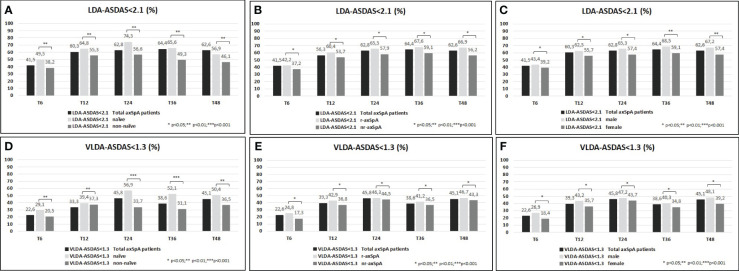
**(A–F)** ASDAS <2.1 (percentage, %) of the overall study population and after their subdivision in two groups according to lines of biological treatment (LoBT) (naïve/non-naïve) **(A)**, axSpA subtype (r-axSpA/nr-axSpA) **(B)**, and sex (male/female) **(C)**. ASDAS <1.3 (percentage, %) of the overall study population and after their subdivision in two groups according to lines of biological treatment (LoBT) (naïve/non-naïve) **(D)**, axSpA subtype (r-axSpA/nr-axSpA) **(E)**, and sex (male/female) **(F)**.

A higher proportion of the study population achieved VLDA and LDA: 39.8%/78.3% and 51.1%/82.3% reached VLDA/LDA-BASDAI at T24 and at T48, respectively (**Figures 1A–D**). Moreover, 45.8%/62.8% and 45.1%/62.6% likewise reached VLDA/LDA-ASDAS at T24 and at T48, respectively (**Figures 2A–D**). We also ascertained the number of patients who achieved VLDA/LDA according to previous LoBT (naïve/non-naïve), axSpA subtype (r-axSpA/nr-axSpA), and sex (male/female).

At T48, naïve patients achieved VLDA/LDA-BASDAI states (**Figures 1A–D**) and VLDA/LDA-ASDAS states (**Figures 2A–D**) in a higher proportion than non-naïve patients. As shown in **Figures 1C–F**, male patients achieved VLDA/LDA-BASDAI states in a higher proportion than female individuals, and similarly for VLDA/LDA-ASDAS states (**Figures 2C–F**). At T48, more r-axSpA patients than nr-axSpA patients achieved VLDA-BASDAI (**Figure 1E**) and VLDA/LDA ASDAS states (**Figures 2B–E**). No differences were observed in LDA–BASDAI achievement in relation to the axSpA subtype (**Figure 1B**).

The proportion of patients undergoing treatment with csDMARDs decreased steadily from T0 (16.5%, *n* = 45) to T6 (18.0%, *n* = 46), T12 (18.3%, *n* = 43), T24 (14.1%, *n* = 27), T36 (8.4%, *n* = 13), and T48 (7.8%, *n* = 7). Similarly, patients treated with GCs decreased from T0 (13.2%, *n* = 36) to T6 [8.6% (*n* = 22)], T12 [8.5% (*n* = 20)], T24 [5.8% (*n* = 11)], T36 [5.2% (*n* = 8)], and T48 [5.6% (*n* = 5)]. We also observed a marked reduction in NSAID intake from T0 (58.8%, *n* = 160) to T6 (39.8%, *n* = 102), T12 (37.0%, *n* = 87), T24 (32.5%, *n* = 62), T36 (29.2%, *n* = 45), and T48 (23.3%, *n* = 21). Throughout the follow-up, we only found a greater reduction of patients taking csDMARDs and GCs in naïve patients than in non-naïve patients (4.8%, *n* = 2 vs. 10.4%, *n* = 5 and 0%, *n* = 0 vs. 10.4%, *n* = 5 at T48, respectively), whereas the percentage of patients taking NSAIDs showed a comparable decrease between naïve vs. non-naïve patients (20.8%, *n* = 10 vs. 26.2%, *n* = 11 at T48).

### 3.3 Drug retention rate

**Figure 3:**
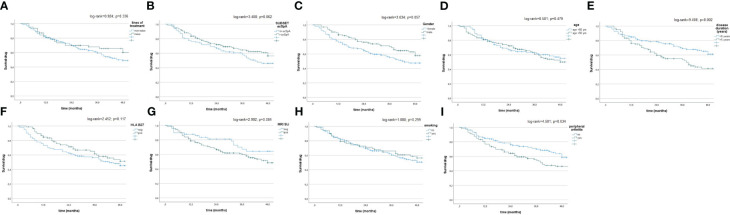
**(A–I)** Drug survival in the overall study population and after their subdivision in two groups, according to lines of biological treatment (LoBT) (naïve/non-naïve) **(A)**, axSpA subtype (r-axSpA/nr-axSpA) **(B)**, sex (male/female) **(C)**, age (>50 years old vs <50 years old) **(D)**, disease duration (<5 years vs. >5 years) **(E)**, HLA B27 (presence vs. absence) **(F)**, active sacroiliitis on MRI (patients with MRI-SIJ+ vs. patients without MRI SIJ+) **(G)**, smoking (yes vs. no) **(H)** and peripheral involvement (patients with peripheral arthritis vs. patients without peripheral arthritis **(I)**.

At T48, DRR was notable (67.4%) in the all patients studied (**Figures 3A–I**), with some dissimilarities considering the disease duration (>5 years vs. <5 years; log-rank = 9.408; *p* = 0.002), concomitant peripheral involvement (subjects with vs. subjects without peripheral arthritis; log-rank = 4.501; *p* = 0.034). The Kaplan–Meier curves did not highlight any differences between naïve vs. non-naïve patients (log-rank = 0.924; *p* = 0.336), male vs. female (log-rank = 3.634; *p* = 0.057), r-axSpA vs. nr-axSpA (log-rank = 3.488; *p* = 0.062), <50 years old vs. >50 years old (log-rank = 0.501; *p* = 0.479), HLA-B27+ patients vs. HLA-B27- patients (log-rank = 2.452; *p* = 0.117), patients with active sacroiliitis on MRI vs. patients without active sacroiliitis on MRI (log-rank = 2.992; *p* = 0.084), and smoking vs. non-smoking patients (log-rank = 1.080; *p* = 0.299).

### 3.4 Comorbidities

Of the axSpA population, 81 (29.8%) patients had at least one comorbidity.

The most frequent comorbidities were hypertension (27.9%, *n* = 76), dyslipidemia (21.7%, *n* = 59), fibromyalgia (18.8%, *n* = 51), osteoporosis (15.1%, *n* = 41), thyroid disorders (13.2%, *n* = 36), gastritis, gastric ulcer, or dyspeptic disorders (12.9%, *n* = 35), metabolic syndrome (MetS) (11%, *n* = 30), ischemic heart disease (9.6%, *n* = 26), positive Mantoux TB skin test or QuantiFERON-TB Gold test (8.8%, *n* = 24) without active tuberculosis, previous hepatitis B (8.5%, *n* = 23), liver disease (e.g., steatosis) (7.7%, *n* = 21), hyperuricemia (7.7%, *n* = 21), depression (7.7%, *n* = 21), neurological disorders (e.g., neuropathy) (6.9%, *n* = 19), diabetes type II (5.9%, *n* = 16), pneumopathies (5.9%, *n* = 16), previously eradicated cancer (5.5%, *n* = 15), previous hepatitis C (1.8%, *n* = 5), and kidney failure (0.7%, *n* = 2). The frequency of these comorbidities was described in both naïve and non-naïve patients (**Supplementary Table S1**). A higher prevalence of cardiovascular pathologies, dyslipidemia, gastric disorders, osteoporosis, fibromyalgia, and neurological disorders was described in non-naïve patients.

### 3.5 Safety and discontinuation

Reasons for discontinuation: Safety and tolerability were notable in patients treated with secukinumab (**Table 3**).

**Table 3:** Reasons for discontinuation of all (*n* = 272), naïve (*n* = 84), and non-naïve (*n* = 188) axSpA patients.

	Total patients	Naïve	Non-naïve	*P* ^§^
Reasons for discontinuation	104 (38.2%)	31 (36.9%)	73 (38.8%)	0.04
Primary loss of effectiveness	17 (6.3%)	5 (5.9%)	12 (6.4%)	0.03
Secondary loss of effectiveness	45 (16.5%)	12 (14.3%)	33 (17.6%)	0.03
Adverse events	34 (12.5%)	11 (13.1%)	23 (12.2%)	ns
Reactions at the injection site or skin manifestations	13 (4.8%)	4 (4.8%)	9 (4.8%)	ns
Leuko-neutropenia	1 (0.4%)	0 (0%)	1 (0.5%)	ns
Dyspnea	1 (0.4%)	0 (0%)	1 (0.5%)	ns
Hypertransaminasemia	1 (0.4%)	0 (0%)	1 (0.5%)	ns
Gastrointestinal disorders (nausea, diarrhea, abdominal pain)	2 (0.7%)	1 (1.2%)	1 (0.5%)	ns
IBD flare-up	1 (0.4%)	0 (0%)	1 (0.5%)	ns
Severe recurrent infections	7 (2.6%)	2 (2.4%)	5 (2.7%)	ns
Fever, arthromyalgia, asthenia	3 (1.1%)	1 (1.2%)	2 (1.1%)	ns
Onset of new cancer	5 (1.8%)	1 (1.2%)	4 (2.1%)	ns
Other reasons for drop-out	8 (2.9%)	2 (2.4%)	6 (3.2%)	ns
Pregnancy	0 (0.0%)	0 (0.0%)	0 (0.0%)	ns
Non-compliance	1 (0.4%)	1 (1.2%)	0 (0.0%)	ns
Remission	0 (0.0%)	0 (0.0%)	0 (0.0%)	ns
Lost to follow-up	7 (2.6%)	3 (3.6%)	4 (2.1%)	ns
Infectious events	57 (21.0%)	12 (14.3%)	45 (23.9%)	0.04
COVID-19 infections	7 (2.6%)	3 (3.6%)	4 (2.1%)	0.04
Other non-COVID-19 respiratory tract infection	15 (5.5%)	5 (5.9%)	10 (5.3%)	ns
Oral or vaginal candidiasis	10 (3.7%)	3 (3.6%)	7 (3.7%)	ns
Labial herpetic infections	7 (2.6%)	2 (2.4%)	5 (2.7%)	ns
Herpes zoster	4 (1.5%)	1 (1.2%)	3 (1.6%)	ns
Gastroenteritis or diverticulitis	4 (1.5%)	1 (1.2%)	3 (1.6%)	ns
Urinary tract infections	8 (2.9%)	2 (2.4%)	6 (3.2%)	ns
Erysipelas or skin/soft tissue infections	2 (0.7%)	1 (1.2%)	1 (0.5%)	ns

Data are expressed as frequency (absolute number and percentage). p < 0.05, naïve vs. non-naïve.

naïve, naïve to TNF inhibitors; non-naïve, TNF inhibitors’ failure.

A total of 104 (38.2%) discontinued treatment with secukinumab throughout the follow-up, mostly owing to primary and secondary loss of effectiveness (17 and 45, respectively). Eight patients dropped out of the observational study spontaneously. Only 34 patients discontinued secukinumab due to AEs: skin rash at the injection site (13) and severe recurrent infections (seven); only one patient presented an IBD flare-up and five patients a new cancer diagnosis [breast cancer (one), follicular thyroid carcinoma (one), colo-rectal adenocarcinoma (two), and prostate cancer (one)]. Blood count and liver and kidney function were monitored, and only two patients exhibited abnormal values.

Infections: A total of 57 mild infections were documented during the study period and were resolved after oral antimicrobial treatment, without hospitalization or drug discontinuation. Only seven patients developed severe infections, prompting the discontinuation of secukinumab (two bronchopneumonia, one urosepsis due to *E. coli*, two erysipelas or skin/soft tissue infections with sepsis due to *S. aureus* with hospitalization, and two recurrent candidiasis).

There were no discernible differences in safety between naïve and non-naïve patients. However, a slightly elevated frequency of primary/secondary loss of effectiveness was observed in non-naïve patients (**Table 3**).

## 4 Discussion

Secukinumab, a first-in-class IL-17 inhibitor, has consistently demonstrated its efficacy in axSpA across several phase III RCTs as part of a comprehensive development program, including the MEASURE trials (13–25) in ankylosing spondylitis (AS) and the PREVENT trial (26) in nr-axSpA. Real-world evidence is gradually emerging from observational data, surveys, and registries (31–36). Our study contributes to the body of evidence, aligning with recent real-life Italian studies published on AS and axSpA (27–30).

In this study, we conducted an assessment of the effectiveness, safety, and DRR of secukinumab in 272 patients with axSpA in a real-world multicenter cohort followed for up to 4 years. To the best of our knowledge, no other studies have thus far been conducted for such an extended observation period and in such a large national cohort of patients. Furthermore, we examined the impact of previous LoBT, axSpA subtype, and sex on achieving good clinical control of disease activity and on drug survival. This study demonstrates that secukinumab provides rapid and sustained effectiveness for up to 48 months of treatment as evidenced by improvements in various outcomes (e.g., ASDAS and BASDAI) across all the study populations. These findings corroborated and built upon data obtained from other RTCs (13–26) as well as observational and registry RWE studies on secukinumab (27–36). Similarly, secukinumab showed sustained increments in the number of patients who achieved ASAS20 and ASAS40 responses in the MEASURE 2 (14, 15), MEASURE 3 (16), and MEASURE 4 studies (17). Data from the extension phase of the MEASURE 1 study indicate that the proportion of patients meeting ASAS20 and ASAS40 criteria remained consistent over 3, 4, and 5 years of treatment among those continuing to take secukinumab (14, 19, 22, 23). Furthermore, the European Spondyloarthritis Research Network Collaboration (EuroSpA) study demonstrates that patients with axSpA receiving secukinumab experience statistically significant improvements across various disease activity parameters (e.g., BASDAI and ASDAS-CRP), functional status, pain, and fatigue (32). An analysis of the BIOBADASER Spanish Registry reveals that patients with SpA, including AS and nr-axSpA patients, treated with secukinumab exhibit improvement after 1 year of treatment, which is sustained or enhanced after 2 and 3 years, with a slight, albeit not markedly better, response observed in biological-naïve patients, suggesting effectiveness in both naïve and non-responder patients. Similarly, the SERENA study, investigating the real-world effectiveness of secukinumab treatment in patients with active AS and SpA, reports significant improvements in disease activity and physical and mental well-being for up to 2 years in all patients (34, 35). These benefits are more pronounced in biological-naïve patients compared to those with prior TNFi treatment (27–29, 31, 32). Additionally, a significantly reduced NSAID intake was observed in our axSpA population receiving secukinumab. A pooled analysis of a large dataset of patients from the MEASURE 2–4 studies had previously provided evidence of the sustained and long-term NSAID-sparing effect of secukinumab over 4 years of treatment (24).

Another aim of this study was to evaluate the impact of LoBT, axSpA subtype, and sex on achieving remission or LDA in our axSpA population. Regarding the first point, we observed that treatment-naïve patients benefited the most from secukinumab. Disease activity remained low throughout the 4-year study period, with a numerically higher benefit observed in the treatment-naïve group, underscoring an early and sustained response. It is well established that the effectiveness of secukinumab is highest when used as the first biological agent in axSpA patients compared to when used as a second- or third-line treatment as evidenced by various studies (27–30, 38–40). Better PROs and higher rates of VLDA)/LDA, as per ASAS and BASDAI criteria, were observed in treatment-naïve patients compared to non-naïve patients in this study, consistent with findings from other real-life studies (27–30, 38–40). However, LoBT did not appear to influence secukinumab discontinuation at the 4-year mark in our axSpA population, which was consistent with findings from other real-life studies, indicating that LoBT does not significantly affect secukinumab retention rates at 12 or 24 months in axSpA patients (27–30, 32). Therefore, secukinumab can be considered effective both as first-line therapy and in patients in whom multiple treatments have failed.

Regarding axSpA subtype, in this analysis, patients with nr-axSpA presented slightly worse clinical measures, physical functioning, and inflammatory activity, which were likely due to the complexity and heterogeneity of this disease, often associated with other SpA features such as peripheral arthritis and extra-articular manifestations. However, the non-radiographic subtype did not appear to influence the persistence of effectiveness. These findings were expected, as secukinumab has been approved for use in the treatment of nr-axSpA in Europe and the USA, based on results from the PREVENT phase III study demonstrating significant and sustained improvement in signs and symptoms of patients with nr-axSpA through 52 weeks (26).

In our study population, male patients achieved better disease control than female ones. The male patients had higher rates of VLDA and LDA achievement than female patients, though the drug retention rates did not vary significantly, indicating consistent secukinumab efficacy over time regardless of sex (29, 39–43). While sex differences in axSpA prevalence and phenotypes are observed in many studies (44), most RCTs were not designed or powered to investigate sex differences or demonstrate treatment responses by sex (13, 26). Previous pooled efficacy analyses of TNFi treatment reported sex differences (10, 31, 45), with notably lower levels of therapeutic response and treatment adherence in female patients (29, 46–48), despite comparable or worse disease burden at baseline in female patients compared to male patients. An important observation is the increasing number of women diagnosed with axSpA over the last decades (44, 47). Moreover, in axSpA, the male-to-female ratio tends to decrease with disease onset after age 40 and the identification of nr-axSpA. Women are known to exhibit poorer responses to TNFi compared to men in SpA, while the influence of sex in IL-17i responses appears more controversial (43). In AS, male patients more frequently achieved inactive disease (ASDAS-ID response) in a *post-hoc* pooled analysis of all MEASURE trials, although no other efficacy outcomes were substantially affected by sex (45). Furthermore, real-world evidence with secukinumab suggests that male patients show higher retention rates than female patients in AS and axSpA (27–36). Our study confirms the lesser ability of female patients to achieve inactive disease or LDA with secukinumab after 4 years of follow-up, although drug survival did not substantially differ between the two sexes.

Regarding persistence, in our axSpA population, a 48-month cumulative secukinumab DRR of 67.4% was estimated, with a median duration of 40 months of drug administration. The overall secukinumab DRR was high both in the short term and the long term, as a similar value (75%) was found at 24 months in our previous study (29). Similarly, the 3-year data from the phase 3 MEASURE 2 trial reported high retention rates varying between 76% and 86% in patients with AS (17). In open-label extensions of clinical trials with secukinumab, involving both biologic-naïve patients and those in whom TNFi treatment had failed, 84% of axSpA patients (15) remained on the drug after 5 years of follow-up, a retention rate higher than those obtained in our real-world study (67.4%), which is an expected finding since retention rates tend to be higher in clinical trials than in real-life studies due to the strict inclusion/exclusion criteria and close patient follow-up in the former scenario. To our knowledge, no national multicenter studies with secukinumab that reached 4 years of follow-up under clinical practice conditions have been published, as our study now does. This result is within the range of what has been published in similar real-life studies, which reported overall retention rates of 76% and 66%, respectively (27–36). Similarly, according to previous studies (27–36), inefficacy (22.8%) was found to be the main cause of secukinumab discontinuation in our study.

The assessment of baseline patient and disease characteristics associated with higher biologic retention may aid in identifying the most appropriate patient profile to achieve better long-term efficacy and safety. Another objective of this work was to identify the patient profile associated with better secukinumab survival. Prior exposure to LoBT, sex, and axSpA subtype did not influence the DRR. Additional analyses conducted in different subgroups of patients regarding age, smoking habits, and the presence *versus* absence of HLA-B27 and sacroiliitis on MRI did not highlight differences in the DRR.

In contrast to TNFi, in our study, secukinumab retention was not associated with patient age, sex, HLA-B27 positivity, or radiographic status, results that were similarly reported by other studies (32–36). Overall, secukinumab is effective regardless of previous experience with TNFi. The findings on sex were consistent with the MEASURE studies, where drug survival was comparable between male and female axSpA patients treated with secukinumab over 52 weeks (43). Similarly, in pooled analyses of the MEASURE studies, secukinumab was effective in patients with r-axSpA regardless of their HLA-B27 status (19, 25, 43, 49). Other than the aforementioned factors, baseline patient characteristics did not have a major impact on the overall secukinumab retention in this study, except for disease duration and concomitant peripheral involvement. The reasons that can be hypothesized are as follows: (1) subjects with higher disease duration are mostly multi-failure bDMARDs patients and (2) the presence of other extraspinal manifestations indicates a subtype of disease that is more complex and difficult to treat and to target.

Regarding safety, in general, secukinumab treatment was well tolerated in patients with axSpA. Throughout the treatment period, no new or unexpected safety signals were observed. The incidence of adverse events (AEs) of special interest was low, and no new cases of uveitis were reported; only one inflammatory bowel disease (IBD) flare-up was observed, suggesting a low risk of uveitis or IBD with secukinumab treatment. Additionally, no cases of hepatitis B or tuberculosis reactivations were observed in our cohort. Secukinumab appears to be safe in axSpA patients with latent tuberculosis infection (LTBI), even in those not receiving anti-TB prophylaxis, which was consistent with a pooled analysis of data across studies in various indications (including the MEASURE 1, 2, 3, and 4 studies), which showed no active cases of TB (50, 51).

The safety profile was consistent with the established safety profile across approved indications and what has been previously reported in RCTs and their long-term extension studies (50–53). An integrated safety analysis, based on data from 21 RCTs with secukinumab, including MEASURE 1, 2, and 3 (*n* = 794), along with post-marketing surveillance data (50–52), reported that the exposure-adjusted incidence rate (EAIR) of any AE was 140.1 per 100 patient-years and of any serious AE was 6.3 per 100 patient-years (50–52). The most common AEs were viral upper respiratory tract infection, headache, diarrhea, and urinary tract infection (50), consistent with our findings. Similarly, 5-year data across a range of indications show a low rate of malignancy in patients receiving secukinumab (54, 55); the EAIR was 0.85 per 100 patient-years, and the observed number of malignancies in our study (*n* = 5) was comparable to the expected number (54).

The strength of this study lies in the fact that its findings complement those of clinical trials. Real-world evidence (RWE) studies offer valuable insights into predictive factors for the effectiveness, safety, and survival of secukinumab in a heterogeneous Italian patient population with various clinical SpA characteristics, a perspective not commonly addressed in RCTs. Moreover, the study enrolled a larger cohort of patients than previous studies and followed them for an extended duration.

However, this study has limitations. Firstly, due to the retrospective collection of patient data solely from available sources, inherent data gaps were unavoidable; retrospective data collection carries a risk of bias due to a lack of standardization. Secondly, a small proportion of patients received secukinumab as first-line treatment, while a larger number received it as second-line or later treatment. Thirdly, radiographic and imaging assessments during the 48-month follow-up were limited. Future studies assessing secukinumab use in axSpA across different regions and countries are warranted.

In conclusion, this real-world study showed a 67.4% retention rate of secukinumab at 4 years of treatment with an acceptable safety profile in axSpA patients, regardless of the length of treatment break, axSpA subtype, or sex. We demonstrated that secukinumab rapidly improves disease activity in axSpA patients, resulting in high persistence rates at 48 months of follow-up. Drug survival reflects treatment safety, effectiveness, and tolerability, serving as a marker of therapeutic success. Finally, our study indicates that secukinumab use in clinical practice aligns with the latest ASAS/EULAR recommendations for axSpA management, positioning IL-17 inhibitors as one of the primary biological disease-modifying antirheumatic drug (bDMARD) options in current practice.

## Data availability statement

The raw data supporting the conclusions of this article will be made available by the authors, without undue reservation.

## Ethics statement

Patients’ written consents were obtained according to the Declaration of Helsinki, when they were first entered into the database for treatment. The Ethics Committee’s approval was obtained from all participating centers (approval no. 23943), as well as the written informed consent for the anonymous use of personal data from every patient, in compliance with Italian Legislative Decree 196/2003. The studies were conducted in accordance with the local legislation and institutional requirements. The participants provided their written informed consent to participate in this study.

## Author contributions

RR: Conceptualization, Data curation, Methodology, Supervision, Validation, Visualization, Writing – original draft, Writing – review & editing. ML: Conceptualization, Data curation, Formal analysis, Methodology, Supervision, Validation, Visualization, Writing – original draft, Writing – review & editing. MC: Data curation, Supervision, Validation, Writing – original draft, Writing – review & editing. SD: Data curation, Supervision, Validation, Writing – original draft, Writing – review & editing. AM: Data curation, Supervision, Validation, Writing – original draft, Writing – review & editing. CS: Data curation, Supervision, Validation, Writing – original draft, Writing – review & editing. EL: Data curation, Supervision, Validation, Writing – original draft, Writing – review & editing. LS: Data curation, Supervision, Validation, Writing – original draft, Writing – review & editing. ML: Data curation, Supervision, Validation, Writing – original draft, Writing – review & editing. FA: Data curation, Supervision, Validation, Writing – original draft, Writing – review & editing. AC: Data curation, Supervision, Validation, Writing – original draft, Writing – review & editing. MM: Data curation, Supervision, Validation, Writing – original draft, Writing – review & editing. MR: Data curation, Supervision, Validation, Writing – original draft, Writing – review & editing. RF: Data curation, Supervision, Validation, Writing – original draft, Writing – review & editing. GC: Data curation, Supervision, Validation, Writing – original draft, Writing – review & editing. LS: Data curation, Supervision, Validation, Writing – original draft, Writing – review & editing. MF: Data curation, Supervision, Validation, Writing – original draft, Writing – review & editing. AC: Data curation, Supervision, Validation, Writing – original draft, Writing – review & editing. NL: Data curation, Supervision, Validation, Writing – original draft, Writing – review & editing. FR: Data curation, Supervision, Validation, Writing – original draft, Writing – review & editing. MF: Data curation, Supervision, Validation, Writing – original draft, Writing – review & editing. EF: Data curation, Supervision, Validation, Writing – original draft, Writing – review & editing. AD: Data curation, Supervision, Validation, Writing – original draft, Writing – review & editing. RF: Data curation, Supervision, Validation, Writing – original draft, Writing – review & editing. AC: Conceptualization, Data curation, Formal analysis, Methodology, Supervision, Validation, Visualization, Writing – original draft, Writing – review & editing.

## Contributing authors of the Spondyloarthritis and Psoriatic Arthritis SIR Study Group ‘Antonio Spadaro’

Giorgio Amato, MD, Rheumatology Unit, A.O.U. Policlinico S. Marco, Catania, Sicilia, Catania, Italy; Valentina Picerno, MD, PhD, Rheumatology Institute of Lucania (IReL), San Carlo Hospital of Potenza and Madonna delle Grazie Hospital of Matera, Potenza, Basilicata, Italy; Emanuela Praino, MD, Rheumatology Unit, ASL BT Andria – DSS4 Barletta, Italy, Barletta-Andria-Trani, Puglia, Italy; Giovanni Striani, MD, Rheumatology Unit, Department of Medicine DIMED, University of Padova, Padova, Veneto, Italy.

## The members of the “Spondyloarthritis and Psoriatic Arthritis Study Group—A. Spadaro” who collaborated to this study are as follows

Roberta Ramonda, Prof, MD, PhD, Rheumatology Unit, Department of Medicine DIMED, University of Padova, Padova, Veneto, Italy; Maria Sole Chimenti, Prof, MD, PhD, Rheumatology, allergology and clinical Immunology, Department of Systems Medicine, University of Rome Tor Vergata, Rome, Lazio, Italy; Salvatore D’Angelo, Prof, MD, PhD, Rheumatology Institute of Lucania (IReL), San Carlo Hospital of Potenza and Madonna delle Grazie Hospital of Matera, Potenza, Basilicata, Italy; Antonio Marchesoni, Prof, MD, PhD, MD, PhD, Rheumatology, Humanitas San Pio X, Milan, Lombardia, Italy; Carlo Selmi, Prof, MD, PhD, Rheumatology and Clinical Immunology, Humanitas Clinical and Research Center IRCCS, Rozzano, Milan, Lombardia, Italy, Department of Biomedical Sciences, Humanitas University, Pieve Emanuele, Milan, Italy; Ennio Lubrano, Prof, MD, PhD, Academic Rheumatology Unit, Dipartimento di Medicina e Scienze per la Salute “Vincenzo Tiberio”, University of Molise, Campobasso, Molise, Italy; Leonardo Santo, MD, Rheumatology Unit, ASL BT Andria – DSS4 Barletta, Italy, Barletta-Andria-Trani, Puglia, Italy; Michele Maria Luchetti Gentiloni, Prof, MD, PhD, MD, Department of Clinical and Molecular Sciences, University Hospital of the Marche Region, Ancona, Marche, Italy; Fabiola Atzeni, Prof, MD, PhD, MD, Rheumatology Unit, University of Messina, Messina, Sicilia, Italy; Alberto Cauli, Prof, MD, PhD, Rheumatology Unit, Department of Medical Sciences, AOU and University of Cagliari, Monserrato, Sardegna, Italy; Maria Manara, MD, PhD, Department of Rheumatology, ASST Gaetano Pini-CTO, Milan, Lombardia, Italy; Maurizio Rossini, Prof, MD, PhD, Rheumatology Unit, Department of Medicine, AOUI University of Verona, Verona, Veneto, Italy; Andrea Doria, Prof, MD, PhD, Rheumatology Unit, Department of Medicine DIMED, University of Padova, Padova, Veneto, Italy; Rosario Foti, Prof, MD, PhD, Rheumatology Unit, A.O.U. Policlinico S. Marco, Catania, Sicilia, Catania, Italy; Antonio Carletto, MD, Rheumatology Unit, Department of Medicine, AOUI University of Verona, Verona, Veneto, Italy; Antonio Carriero, MD, PhD, Rheumatology Institute of Lucania (IReL), San Carlo Hospital of Potenza and Madonna delle Grazie Hospital of Matera, Potenza, Basilicata, Italy; Mariagrazia Lorenzin, MD, PhD, Rheumatology Unit, Department of Medicine DIMED, University of Padova, Padova, Veneto, Italy; Roberta Foti, MD, Rheumatology Unit, A.O.U. Policlinico S. Marco, Catania, Sicilia, Catania, Italy; Giacomo Cozzi, MD, Rheumatology Unit, Department of Medicine DIMED, University of Padova, Padova, Veneto, Italy; Laura Scagnellato, MD, Rheumatology Unit, Department of Medicine DIMED, University of Padova, Padova, Veneto, Italy; Mario Ferraioli, MD, PhD, Rheumatology, allergology and clinical Immunology, Department of Systems Medicine, University of Rome Tor Vergata, Rome, Lazio, Italy; Nicoletta Luciano, MD, Rheumatology and Clinical Immunology, Humanitas Clinical and Research Center IRCCS, Rozzano, Milan, Lombardia, Italy; Francesca Ruzzon, MD, Rheumatology Unit, Department of Medicine, AOUI University of Verona, Verona, Veneto, Italy; Mauro Fatica, MD, Rheumatology, allergology and clinical Immunology, Department of Systems Medicine, University of Rome Tor Vergata, Rome, Lazio, Italy; Elena Fracassi, MD, PhD, Rheumatology Unit, Department of Medicine, AOUI University of Verona, Verona, Veneto, Italy; Giorgio Amato, MD, Rheumatology Unit, A.O.U. Policlinico S. Marco, Catania, Sicilia, Catania, Italy; Valentina Picerno, Rheumatology Institute of Lucania (IReL), San Carlo Hospital of Potenza and Madonna delle Grazie Hospital of Matera, Potenza, Basilicata, Italy; Emanuela Praino, MD, Rheumatology Unit, ASL BT Andria – DSS4 Barletta, Italy, Barletta-Andria-Trani, Puglia, Italy; Giovanni Striani, MD, Rheumatology Unit, Department of Medicine DIMED, University of Padova, Padova, Veneto, Italy.

## Glossary

**Table:**

SpA	spondyloarthritis
axSpA	axial spondyloarthritis
r-axSpA	radiographic axSpA
nr-axSpA	non-radiographic axSpA
IBP	inflammatory back pain
SIJ	sacroiliac joints
MRI	magnetic resonance imaging
IBD	inflammatory bowel disease
HLA	human leukocyte antigen
ASAS	Assessment of Spondyloarthritis International Society
EULAR	European Alliance of Associations for Rheumatology
ACR	American College of Rheumatology
NSAIDs	non-steroidal anti-inflammatory drugs
bDMARDs	biological disease-modifying anti-rheumatic drugs
tsDMARDs	targeted synthetic disease-modifying anti-rheumatic drugs
TNFi	tumor necrosis factor inhibitors
IL-17i	IL-17 inhibitors
csDMARDs	conventional synthetic disease-modifying anti-rheumatic drugs
GCs	glucocorticosteroids
RCTs	randomized controlled trials
RWE	real-world evidence
LoBT	lines of bDMARDs treatment
LDA	low disease activity
VLDA	very low disease activity
DRR	drug retention rate
SIR	Italian Society of Rheumatology
PROs	patient-reported outcomes
VAS	Visual Analogue Scale
VAS pain	Visual Analogue Scale of pain
VAS-GH	Visual Analogue Scale Global Health
VAS-Ph	Visual Analogue Scale Physician Health
HAQ-S	Assessment Questionnaires Modified for Spondyloarthritis
BASFI	Bath Ankylosing Spondylitis Functional Index
BASDAI	Bath Ankylosing Spondylitis Disease Activity Index
ASDAS	Ankylosing Spondylitis Disease Activity Score
LEI	Leeds Enthesitis Index
BASMI	Bath Ankylosing Spondylitis Metrology Index
ESR	erythrocyte sedimentation rate
CRP	C-reactive protein
T/SJ	tender/swollen joints
MetS	metabolic syndrome
AEs	adverse events
IQR	interquartile range.
