# Supplementary material for: Four-year real-world experience of secukinumab in a large Italian cohort of axial spondyloarthritis
Source: Front Immunol. 2024 Jul 15;15:1435599. doi: 10.3389/fimmu.2024.1435599 (PMC11284505; doi:10.3389/fimmu.2024.1435599)
Supplement: Supplementary file 1 [file Table_1.docx]

**Supplementary Table 1. Comorbidities of all (n=272), naïve (n=84) and non-naïve (n=188) axSpA patients.**

| **Comorbidities** | ***Total patients*** | ***naïve*** | ***non-naïve*** | ***p*** |
| --- | --- | --- | --- | --- |
| **metabolic syndrome** | 30 (11.0%) | 11 (13.1%) | 19 (10.1%) | *ns* |
| **hypertension** | 76 (27.9%) | 18 (21.4%) | 58 (30.9%) | *0.03* |
| **ischemic heart disease** | 26 (9.6%) | 5 (5.9%) | 21 (11.2%) | *0.04* |
| **dyslipidemia** | 59 (21.7%) | 14 (16.7%) | 45 (23.9%) | *0.04* |
| **hyperuricemia** | 21 (7.7%) | 5 (5.9%) | 16 (8.5%) | *0.048* |
| **type II diabetes** | 16 (5.9%) | 5 (5.9%) | 11 (5.9%) | *ns* |
| **gastritis, gastric ulcer or dyspeptic disorders** | 35 (12.9%) | 7 (8.3%) | 28 (14.9%) | *0.04* |
| **liver disease (e.g. steatosis)** | 21 (7.7%) | 7 (8.3%) | 14 (7.4%) | *ns* |
| **pneumopathies** | 16 (5.9%) | 5 (5.9%) | 11 (5.9%) | *ns* |
| **thyroid disorder** | 36 (13.2%) | 12 (14.3%) | 24 (12.8%) | *ns* |
| **osteoporosis** | 41 (15.1%) | 7 (8.3%) | 34 (18.1%) | *0.03* |
| **kidney failure** | 2 (0.7%) | 1 (1.2%) | 1 (0.5%) | *ns* |
| **depression** | 21 (7.7%) | 5 (5.9%) | 16 (8.5%) | *0.04* |
| **fibromyalgia** | 51 (18.8%) | 12 (14.3) | 39 (20.7%) | *0.04* |
| **neurological disorders (such as neuropathy)** | 19 (6.9%) | 4 (4.8%) | 15 (7.9%) | *0.047* |
| **positive Mantoux TB skin test or QuantiFERON-TB Gold test** | 24 (8.8%) | 10 (11.9%) | 14 (7.4%) | *ns* |
| **previous hepatitis B** | 23 (8.5%) | 9 (10.7%) | 14 (7.4%) | *ns* |
| **previous hepatitis C** | 5 (1.8%) | 2 (2.4%) | 3 (1.6%) | *ns* |
| **previous eradicated cancer** | 15 (5.5%) | 3 (3.6%) | 12 (6.4%) | *0.04* |

**Legend:** Data are expressed as frequency (absolute number and percentage). *p* <0.05 naïve vs non-naïve.

naïve = naïve to TNF inhibitors; non-naïve = TNF inhibitors failure.
